# Integrating omics and functional data via representation learning to prioritize candidate genes for pleiotropic effect in dairy sheep

**DOI:** 10.1093/pnasnexus/pgaf361

**Published:** 2025-11-13

**Authors:** Pablo Augusto de Souza Fonseca, Aroa Suárez-Vega, Laura Casas, Hector Marina, Beatriz Gutiérrez-Gil, Juan Jose Arranz

**Affiliations:** Instituto de Ganadería de Montaña (CSIC-Univ. de León), Finca Marzanas, Grulleros, 24346 León, Spain; Dpto. Producción Animal, Facultad de Veterinaria, Universidad de León, Campus de Vegazana s/n, 24007 León, Spain; Dpto. Producción Animal, Facultad de Veterinaria, Universidad de León, Campus de Vegazana s/n, 24007 León, Spain; Assafe, National Assaf Sheep Breeders Association, Granja Florencia, Toro, 49800 Zamora, Spain; Dpto. Producción Animal, Facultad de Veterinaria, Universidad de León, Campus de Vegazana s/n, 24007 León, Spain; Dpto. Producción Animal, Facultad de Veterinaria, Universidad de León, Campus de Vegazana s/n, 24007 León, Spain; Dpto. Producción Animal, Facultad de Veterinaria, Universidad de León, Campus de Vegazana s/n, 24007 León, Spain

**Keywords:** dairy sheep, pleiotropy, genetic correlation, machine learning, omics integration

## Abstract

The global demand for improved productivity, sustainability, welfare, and quality in livestock production presents significant challenges for breeders. Understanding trait correlations, often driven by pleiotropy, is essential for simultaneously improving traits of economic interest. Integrating multi-omics data and functional annotations can improve the disentangling of biological processes underlying the pleiotropic effect. Network-based machine learning (ML) models offer a robust solution for this integration. This study estimated gene-level *P*-values for pleiotropic effects using two phenotypic datasets: (i) Trait_GWAS, with phenotypic values of 12 traits covering milk production, composition, cheeseability, and mastitis resistance; and (ii) EBV_GWAS, with estimated breeding values for five similar traits, excluding cheeseability. Weighted gene co-expression networks (WGCNs) were constructed from milk somatic cell transcriptomics of Assaf ewes. Gene-term networks were built from gene ontology, metabolic pathways, and quantitative trait loci annotation for the genes in the WGCN. These networks were processed through a representative learning pipeline to create a latent vector representing gene importance. A hierarchical model integrated gene-level *P*-values and the latent vector, generating posterior probabilities of association for each gene. Significant results included 14 and 111 genes for Trait_GWAS and EBV_GWAS, respectively, with three shared genes (*PHGDH*, *SLC1A4*, and *CSN3*). Prioritized genes were linked to biological processes such as amino acid transport, lipid metabolism, mammary gland development, and immune regulation, often involving multiple biological functions. This reinforces the potential pleiotropic role of these genes. These findings highlight the utility of network-based ML models for prioritizing candidate genes with pleiotropic effects on milk, cheese, and health-related traits in dairy sheep.

Significance Statement This study addresses the challenge of improving productivity and sustainability in dairy sheep by identifying genes with pleiotropic effects on milk, cheese, and health-related traits. Significant genes were prioritized using multi-omics integration and network-based machine learning (ML) models based on their associations with critical biological processes, including amino acid transport, lipid metabolism, and immune regulation. These findings demonstrate the potential of ML approaches to unravel complex genetic relationships and guide targeted breeding strategies.

## Introduction

The increase in the world's population leads to a rise in the demand for food. Consequently, creating a scenario where the livestock sector needs to improve productivity when simultaneously improves the sustainability, welfare, and quality standards, resulting in a challenging scenario for breeders (1). In the context of the dairy sheep industry in Spain, the main selection goals are focused on the milk yield and solids content. Nevertheless, a high demand for cheese production and reduced somatic cell contents in the milk is also observed (2, 3). Among the wide range of dairy sheep breeds in Spain, the Assaf breed stands out, and several studies have been conducted to identify the genetic components associated with different production-related traits (4–6). Among these studies, moderate-to-high genetic correlations were identified among milk technological traits, such as milk solid content and cheese-ability-related traits, and somatic cell counts in the Assaf breed (5). A better understanding of the genetic correlation among traits is one of the key steps to achieving simultaneous selection of different traits. One of the main causes of the genetic correlation between traits is pleiotropy, the phenomenon observed when a genetic locus is associated with two or more traits (7). Therefore, the identification of genetic variants (and the genes harboring such variants) is a useful approach to target the biological processes associated with the genetic correlation between traits. Additionally, identifying these potential causal mutations for the pleiotropic effect across can allow a more efficient simultaneous selection for multiple traits during the genetic selection process. However, the proper identification of a pleiotropic effect can be challenging. Based on the genetic mechanisms behind the pleiotropic effect, it is possible to classify the pleiotropy in various classes that will act through different mechanisms (8–10). The investigation of the contribution of a locus associated with a pleiotropic effect into different levels of biological information can improve the classification of its effect. Therefore, integrating multi-omics data and different functional annotation sources emerges as a useful approach. Nevertheless, a successful multi-omics integration pipeline must handle multidimensionality problems efficiently, nonlinear relationships among variables, and multicollinearity problems, among other noise sources (11). In this context, machine learning (ML) models might be a useful alternative to deal with these issues due to their flexible and nonparametric behavior and the ability to deal with multidimensionality. Several network-based ML models have already been described for integrating omics data to prioritize candidate genes (12–15). Among them, the use of representative learning to perform dimensionality reduction from gene networks stands out as a useful approach (15, 16). Indeed, network-based representative learning has been shown to be an effective method for the prioritization of candidate genes for complex traits in genome-wide association studies (GWASs) using different sources of functional information in humans (15, 16). Therefore, the objective of this study was to construct a pipeline for integrating multi-omics data and functional information, based on network embedding and representative learning, to prioritize candidate genes with pleiotropic effects observed between production and health traits in sheep.

## Results

### Genotypes, imputation, GWASs, and pleiotropic effect

Regarding the phenotypic data, the first dataset was composed of 12 phenotypes (Trait_GWAS). A complete description of the phenotyping process for those traits is available in Marina et al. (6). Briefly, five milk production and composition traits, including milk yield (kg), fat percentage (%), fat yield (kg), protein percentage (%), and protein yield (kg), and the logarithmic base 10 transformations of the milk somatic cell counts (logSCCs, cells/mL) were evaluated. Additionally, six cheese-making-related traits, such as rennet clotting time (min), logarithmic base 10 transformations of the time necessary for the curd to reach 20 mm or curd-firming time (logK20, min), and the curd firmness at 30 and 60 min after rennet addition (A30 and A60, mm), individual laboratory cheese yield (g/10 mL of milk), and the individual laboratory dried curd yield (ILDCY, g/10 mL), were also measured for the same animals. The dataset composed of 3,459 Assaf dairy ewes included the estimated breeding values (EBVs) for five traits (EBV_GWAS): combined index for production (50% genetic value for fat + 50% genetic value for protein, ICOp), genetic value for fat at 150 days (GVFAT), genetic value for protein at 150 days (GVPROT), genetic value for milk production at 150 days (GV150), and genetic value for mastitis resistance (GVMR). In total, 493,938 and 515,544 markers were available after imputation for the Trait_GWAS and EBV_GWAS datasets, respectively. The *P*-values obtained for each marker in the estimation of the pleiotropic effect using GWAS summary statistics for each dataset are available in Table S1. The gene-level *P*-values for the pleiotropic effect for the traits evaluated on the Trait_GWAS and EBV_GWAS are shown in Table S2. The Manhattan plots showing the most significant genes in each chromosome for each dataset are available in Fig. S1. The evaluation of the distribution of the *P*-values at the gene level suggested an inflation in the number of significant genes with a potential pleiotropic effect: 1,405 genes for Trait_GWAS and 17,973 for EBV_GWAS. Therefore, this indicates that the direct interpretation of these values might not be informative for selecting candidate genes for the pleiotropic effect on milk production traits in the Assaf breed. Additionally, it is important to mention that 595 and 7,433 significant genes from Trait_GWAS and EBV_GWAS were not included in embedding gene network steps due to the absence of an assigned gene symbol.

### Network embedding and enrichment analyses

**Fig. 1.**
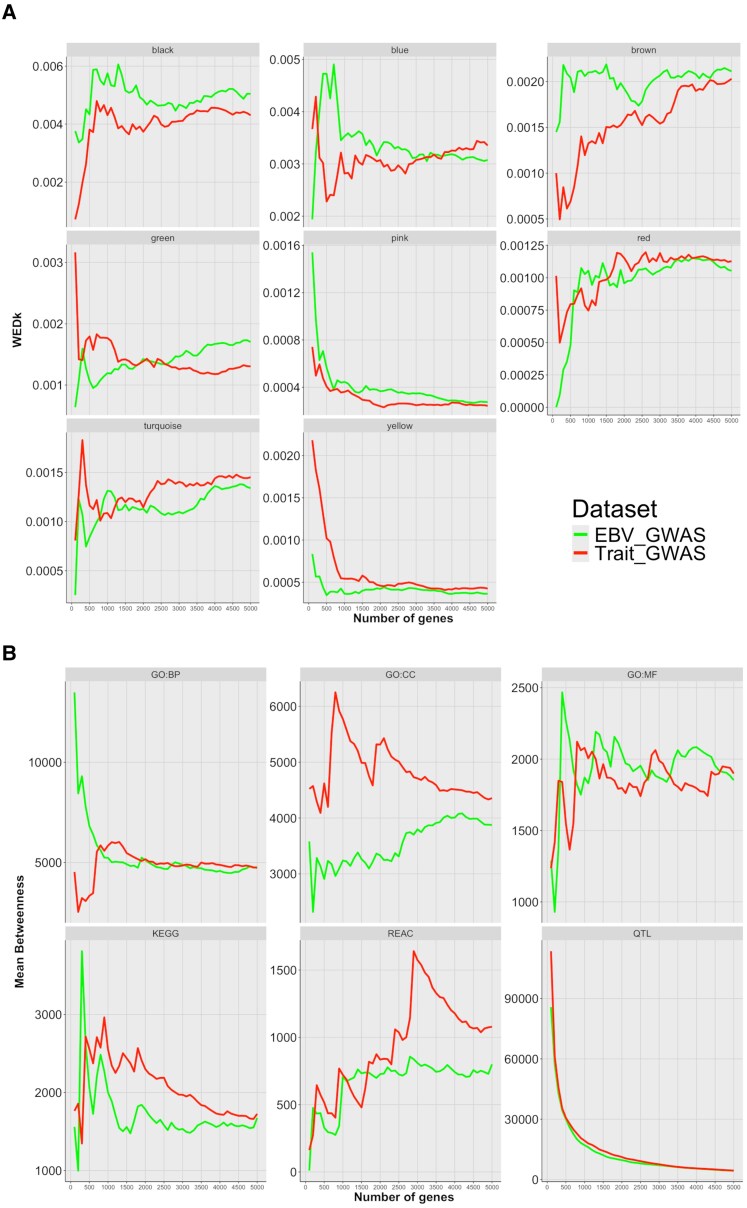
Mean weighted edge density (WED_K_) (A) and betweenness (B) for the top *K* gene (*K* ranges from 100 to 5,000 with step size 100) based on the decreasing sorted list of *P*-values. The green and red lines represent the mean value obtained for the EBV_GWAS and Trait_GWAS, respectively.

The weighted gene co-expression network (WGCN) analysis resulted in the identification of eight modules of co-expressed genes. The list of genes assigned to each module is available in Table S3. The list of enriched GO terms, metabolic pathways, and quantitative trait loci (QTL) identified and used to create the incidence matrices is available in Table S4. The distribution of the weighted edge density of the top *K* gene (WED_K_) values for the WGCN suggested that for the modules pink and yellow, a higher interaction is observed for the genes with the smallest *P*-values (Fig. 1A). For the other modules, a noisier pattern is observed. The enrichment results for GO terms and KEGG pathways obtained for the yellow and pink modules are shown in Figs. S2 and S3. In general, the yellow module presented an association with the metabolism of amino acids, ncRNA, and tRNAs, energy metabolism, and thermogenesis. On the other hand, the pink module was widely associated with lipid metabolism. The network topology of the betweenness distribution for the networks composed of genes and enriched terms showed a clear decreasing pattern of the betweenness when more genes (with smaller posterior probabilities of association [PPA]) were included in the bins for the QTL network. Therefore, this suggests that the genes with the smallest *P*-values are associated with more enriched QTLs. This pattern was consistent for both datasets, Trait_GWAS and EBV_GWAS (Fig. 1B). In addition, for the EBV_GWAS dataset, the same pattern was observed for the network composed of genes and GO:BP terms (Fig. 1A). Despite a noisier pattern, the network of genes and enriched KEGG pathways indicated the same pattern for both datasets (Fig. 1A).

### Prioritization of candidate genes for pleiotropic effect

**Fig. 2.**
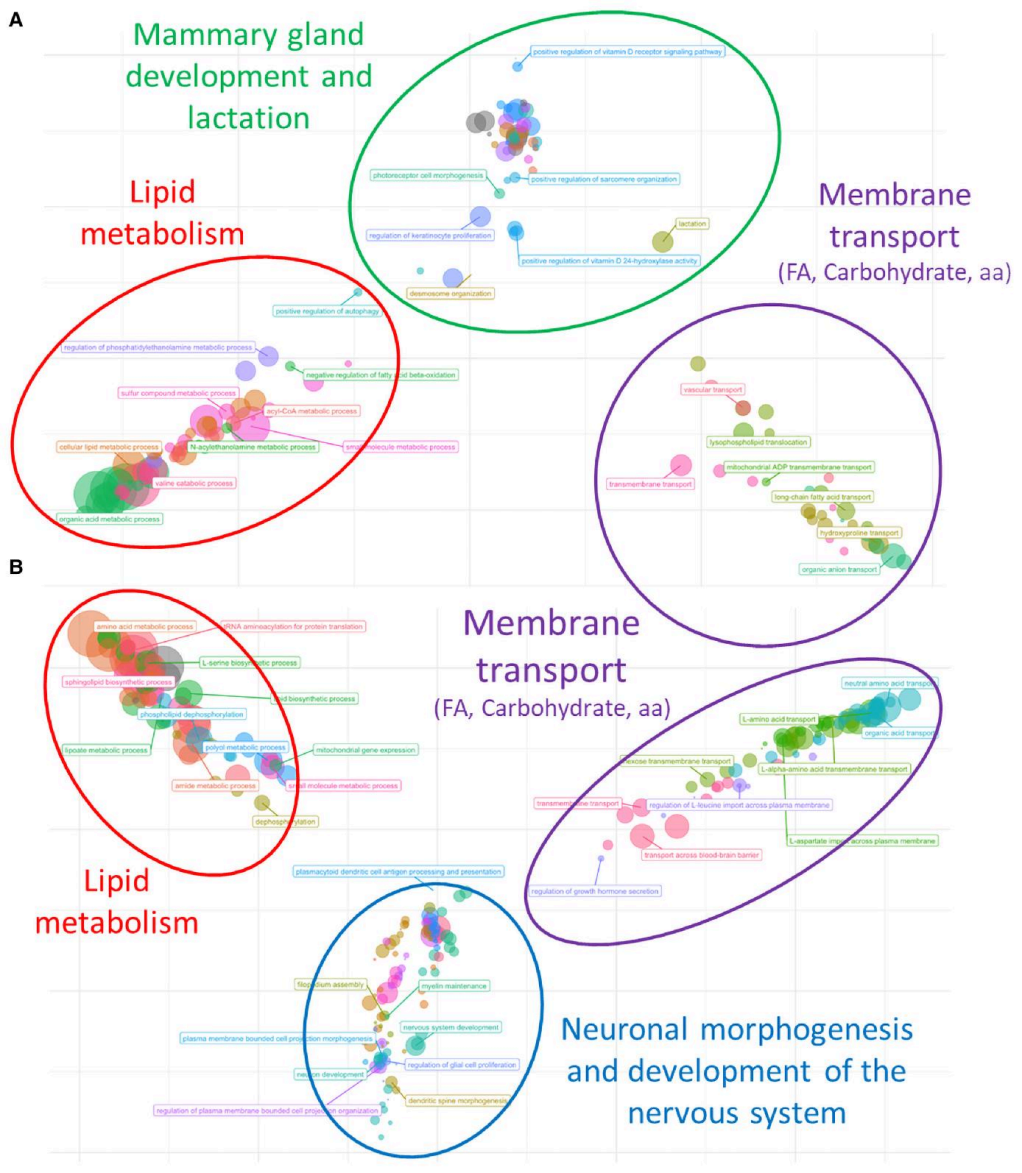
Clusters of enriched terms based on the similarity among terms for the list of prioritized genes identified for Trair_GWAS (A) and EBV_GWAS (B).

The top 1% of the PPA value of each dataset was retained as potential candidate genes for the pleiotropic effect on milk production traits in the Assaf breed. In total, 153 and 149 genes were retained for the Trait_GWAS and EBV_GWAS datasets, respectively. Among these genes, 38 (14.4%) were shared between both datasets. For the genes in the top 1% of the PPA values, 14 and 111 genes were also significant at the gene-level *P*-value for the Trait_GWAS and EBV_GWAS datasets, respectively (Table S5). These genes were considered potential candidates for the pleiotropic effect. Only three genes (*PHGDH*, *SLC1A4*, and *CSN3*) were shared between Trait_GWAS and EBV_GWAS. The complete list of enriched GO terms and metabolic pathways enriched for the prioritized genes is shown in Table S6. The analysis of the GO terms enriched for the 14 prioritized genes for the Trait_GWAS dataset suggests that those genes are associated with three main classes of processes: lipid metabolism, membrane transport, and vitamin D metabolism (Fig. 2). Additionally, genes associated with the regulation of lactation (*CSN3* and *VDR*) and the control of the involution of the mammary gland (*VDR*) were observed. Similarly, with the result obtained for the Trait_GWAS dataset, the prioritized genes for the EBV_GWAS dataset showed an association with lipid metabolism and membrane transport. However, a more specific membrane transport activity could be identified in this case. The prioritized genes for the EBV_GWAS dataset involved with membrane transport activity seem to play roles in the transport of amino acids, such as L-aspartate and L-leucine (Fig. 2B). A cluster of enriched terms associated with neural development was also identified for these genes (Fig. 2B). Indeed, a large proportion of the prioritized genes were associated with enriched terms related to neuronal morphogenesis and nervous system development, as shown in Table S6. Additionally, genes regulating growth hormone secretion are among the prioritized genes for the EBV_GWAS dataset (*KALRN* and *ARHGEF7*). Interestingly, the three prioritized genes shared between both datasets are associated with at least one of the functional classes described before. The *PHGDH* and *SLC1A4* were associated with enriched terms related to the nervous system and amino acid metabolism and transport, while *CSN3* was associated with enriched terms related to membrane transport.

**Fig. 3.**
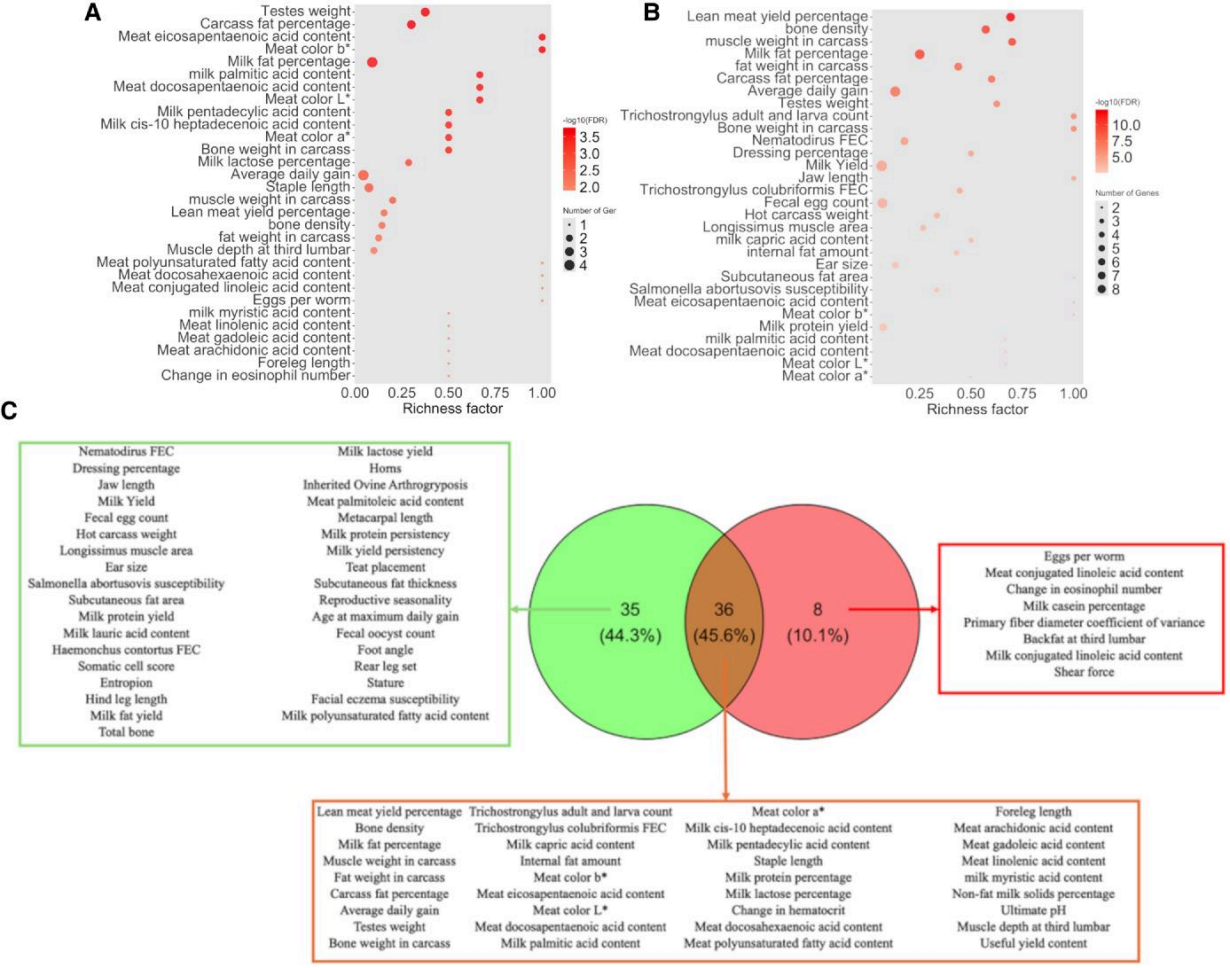
Top 30 enriched QTL identified for the list of prioritized genes obtained for the Trait_GWAS (A) and EBV_GWAS (B) datasets. The area of the red dot represents the number of QTLs annotated for the enriched trait. The color represents the *P*-value adjusted for the false discovery rate in the −log scale, where more intense red colors indicate smaller *P*-values. C) Venn diagram showing the intersection between the enriched QTLs identified for Trait_GWAS (in red, right-hand side) and EBV_GWAS (in green, left-hand side) datasets.

**Fig. 4.**
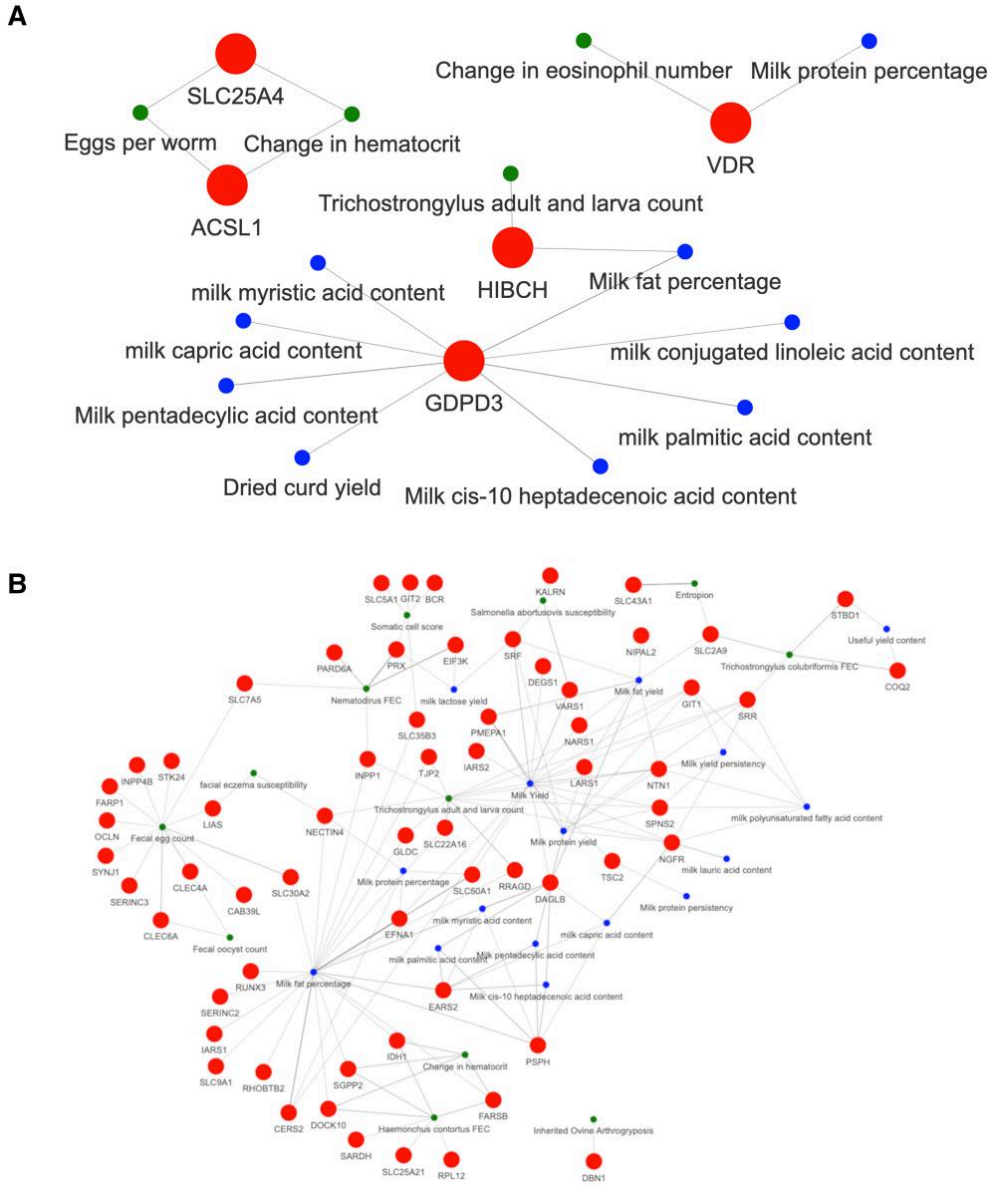
Gene and QTL interaction network. The edges represent a co-location of a gene in a 100-kb window (downstream or upstream) from an enriched QTL identified for the list of prioritized genes obtained for the Trait_GWAS (A) and EBV_GWAS (B) datasets. The red, blue, and green nodes represent the genes, the milk-related QTLs and health-related QTLs, respectively.

To add more biological context to our findings, we downloaded previously reported QTLs in sheep and annotated them within our set of significant genes showing pleiotropic effects and performed a QTL enrichment analysis. The top 30 enriched QTLs for each prioritized gene list are shown in Fig. 3. Additionally, Fig. S4 shows the relationship between the prioritized genes and the enriched QTLs identified in the analyses of the WGCN. The complete lists of annotated and enriched QTLs in the 100 kb interval upstream and downstream from the coordinates of the prioritized genes are available in Tables S7 and S8, respectively. In total, 44 and 71 QTLs were enriched for the list of prioritized genes for the Trait_GWAS and EBV_GWAS datasets. Among these QTLs, 36 were shared between both datasets (Fig. 3C). Among the shared enriched QTLs, several QTLs for the fatty acid content in the milk and meat were included. Additionally, QTLs for protein, lactose, and solids were shared between the datasets. Interestingly, the list of prioritized genes for the EBV_GWAS datasets was associated with several QTLs related to the body structure, such as ear size, jaw length, hind leg length, total bone, teat placement, and stature. Figure 4 shows the relationship between the enriched QTLs from the health- and milk-related types, identified using the list of prioritized genes for the Trait_GWAS and EBV_GWAS.

## Discussion

The discovery of functional candidate genes is crucial to understanding the biological processes behind pleiotropic effects (8–10). Additionally, identifying genes with pleiotropic effects releases the possibility of determining the causality of genetic variants over multiple traits simultaneously. However, scrutinizing the contribution of a specific gene over multiple traits is a complex task that relies on the investigation of its function on multiple biological levels. The fast development of high-throughput omics technologies and the enhancement of public databases, including functional information of genes at different levels, provided the possibility of integrating a wide range of information in statistical models. However, these models must be able to deal with specific challenges raised in multidimensional and variable data, such as nonlinear relationships and multicollinearity. Network embedding and representative learning models are good alternatives for this task due to their ability to reduce the dimensionality of the data, maintain the informativeness, and allow an efficient implementation in other models (15, 16).

This study evaluated the use of network embedding and representative learning to analyze gene networks and generate latent representation vectors. These vectors were then fitted into hierarchical models to create a pleiotropic probability assessment or PPA. These PPA represented the probability of gene associations and their potential pleiotropic effect on production and health traits in the studied populations. The pipeline presented here is adapted from the REGENT pipeline previously described for the prioritization of GWAS results in humans (15). It is important to reinforce that in the Trait_GWAS dataset, the production traits are milk- and cheese-related traits, while the logSCC in the milk represents the health-related trait. On the other hand, for the EBV_GWAS dataset, only EBVs for milk-related production traits and a mastitis resistance marker were available.

### Prioritized genes for the pleiotropic effect observed for the Trait_GWAS dataset

The prioritization pipeline resulted in the detection of 11 genes exclusively prioritized for the Trait_GWAS dataset (*HIBCH*, *VDR*, *PRKD1, MFSD2A*, *ALDH6A1, PTGR2, ACOT6, GDPD3, GRHL1*, *SLC25A4*, and *ACSL1*). The *HIBCH* gene had the smallest gene-level *P*-value and a PPA = 1 for the pleiotropic effect on the Trait_GWAS dataset. The *HIBCH* codifies an enzyme responsible for catalyzing the conversion of 3-hydroxy-isobutyryl-CoA to free 3-hydroxy-isobutyrate, a crucial valine catabolic process (17). Indeed, the levels of 3-hydroxy-isobutyrate in the saliva were suggested to be a reliable biomarker for valine metabolism in humans (18). Regarding health traits in livestock, the 3-hydroxy-isobutyrate was identified as differentially abundant between healthy and hyperketonemic cows (19). The metabolite levels of hydroxy-isobutyrate are also associated with feed efficiency in beef cattle (20, 21). Results from gene silencing assays suggested that *HIBCH* may be a key mediator of lipid metabolism through valine metabolism (22, 23). In the porcine mammary gland, 3-hydroxy-isobutyrate promotes the proliferation of epithelial cells and lipid metabolism (24). In cattle, 3-hydroxy-isobutyrate was identified as a differentially expressed protein between lactating and nonlactating cows (25). Among the prioritized genes in the Trait_GWAS dataset, another gene responsible for the codification of an enzyme responsible for the control of the step in the valine degradation was identified. The *ALDH6A1* codifies the enzyme methylmalonate semialdehyde dehydrogenase, which converts methylmalonate semialdehyde to propionyl-coenzyme A (26).

Mutations of *ALDH6A1* are associated with methylmalonic aciduria in humans (27). Additionally, based on the enrichment analysis, the genome region where *ALDH6A1* is located was previously associated with QTL influencing milk yield in sheep. The *VDR* gene codifies the vitamin D receptor, a key molecule for controlling vitamin D activation and regulating several biological processes, such as cellular proliferation, differentiation, and immune response (28). Among different cellular types, the VDR seems to act in the negative growth regulation of mammary cells (29, 30). In the context of the mammary gland, vitamin D is suggested to have a protective role against mastitis (31–33).

The *GRHL1* gene codifies a transcription factor that, together with VDR, was associated with the differentiation of keratinocytes by the enriched GO terms in the current study. Indeed, this transcription factor seems to play an important role in the differentiation and migration of keratinocytes through the preferential binding on super-enhancers (34). The differentiation and migration of keratinocytes is a crucial process related to the mammary gland development, which acts to form the teat and papillary annulus (35). The teat canal is lined with folds of keratinized skin epidermis, which has antibacterial properties that help avoid pathogenic mastitis microorganisms (36–38).

The prioritized genes *MFSD2A*, *ACOT6*, *PTGR2*, *PRKD1*, *ACSL1*, and *GDPD3* were associated with several enrichment terms related to lipid metabolism. Interestingly, among these genes, *MFSD2A*, *PTGR2*, *ACOT6*, *ACSL1*, and *GDPD3* act by regulating or interacting with the peroxisome proliferator-activated receptor gamma (PPARγ) pathway (39–43). The PPARγ is a crucial gene for the development of the mammary gland in ruminants, controlling biological processes such as lactogenesis, control of inflammation, and adipogenesis (44–47). Among these genes, the functional roles of *ACSL1* and *ACOT6* stand out. *ACSL1* regulates the triglyceride levels and is among the more abundant transcripts in the mammary gland of cows and goats (46, 48, 49). On the other hand, ACOT6 acts on the activation of fatty acids, releasing the corresponding nonesterified fatty acid and coenzyme A (50). Another interesting biological function for the pleiotropic effect in the evaluated trait was associated with the *GDPD3* gene. This gene codifies a phospholipase D enzyme, which has important activities for its application in cheese production (51). In the current study, *GDPD3* showed the largest milk-related enriched QTLs, mainly associated with fat content and dried curd yield. The prioritized gene *PRKD1* was associated with enriched terms of lipid metabolism and keratinocyte differentiation in the current study. Additionally, this gene was previously associated with milk quality traits in Gannan Yak (52). Finally, the prioritized gene *SLC25A4* codifies the solute carrier (SLC) family 25 member 4, which was demonstrated to be negatively expressed within the mitochondria of the mammary gland during the pregnancy-to-lactation transition (53).

### Prioritized genes for the pleiotropic effect observed for the EBV_GWAS dataset

In total, 111 genes were exclusively enriched for the EBV_GWAS dataset. Consequently, discussing all these genes in detail is not viable. Therefore, the discussion will be based on groups of genes with similar functions and some specific functional candidate genes. Around 16% of the prioritized genes for the EBV_GWAS dataset (18 genes) are members of the SLC family. The abundance of SLC transcripts and the regulation of genes that control energy homeostasis are crucial in supporting milk synthesis during the postpartum to lactation transition (52). Some prioritized genes are functional candidate genes for the potential pleiotropic effect observed. The *SLC7A5* codifies a transporter protein independent of Na^+^ and pH responsible for regulating cell growth through the uptake of amino acids (54, 55). In goats, the overexpression of *SLC7A5* is associated with the increase of expression in some important genes for milk production and composition, such as *CSN1S1*, *SCD*, *CEBPB*, and *ACACA* (56). Other genes, such as *SLC30A2*, *SLC1A4*, and *SLC38A3,* were identified as differentially expressed in the lactating mammary gland (57–59). Indeed, in the current study, the *SLC30A2* gene showed the highest betweenness score for the network generated using the list of prioritized genes for EBV_GWAS and the enriched QTLs. Additionally, some of the prioritized SLC genes were associated with >100 enriched terms (*SLC1A2* [124 genes], *SLC1A3* [121 genes], *SLC7A5* [109 genes], and *SLC38A3* [107 genes]). These results reinforce the potential role of the amino acid transport-related genes with the pleiotropic effect observed between production and health traits in dairy sheep.

The enriched terms associated with lipid metabolism were among the three main clusters of biological processes related to the prioritized genes for the EBV_GWAS dataset, comprising 21 genes (∼19%). The *DAGLB* gene is among these genes and showed the second-highest betweenness score for the network generated using the list of prioritized genes for EBV_GWAS and the enriched QTLs. The milk-related enriched QTLs associated with *DAGLB* are mainly enrolled with the control of milk yield, milk fatty acid content, and milk fat percentage. This gene codifies the enzyme diacylglycerol lipase-beta, which is responsible for the catalyzation of the hydrolysis of arachidonic acid–esterified diacylglycerols to produce the principal endocannabinoid (arachidonoyl-glycerol) (60). This process places *DAGLB* as a key metabolic hub in the regulation of proinflammatory responses in macrophages through the lipid signaling network (60, 61). Two ceramide synthases, *CERS2* and *CERS4*, are also among the genes associated with lipid metabolism. Both genes are intrinsically associated with the control of lipid metabolism, cell division, and insulin resistance (62–65). Insulin resistance is a risk factor for many metabolic disorders in dairy cows and sheep (66–69). Another gene related to the synthesis of ceramides, *DEGS1*, was also prioritized for the pleiotropic effect observed in the EBV_GWAS dataset. The *DEGS1*, which codifies the enzyme 4-desaturase, sphingolipid 1, is associated with the synthesis of sphingolipids and adipocyte differentiation (70, 71). Interestingly, *DEGS1* was identified among the most abundant transcripts related to lipid metabolism in three stages of lactation (peak lactation, cessation of milking, and involution) in dairy goats (72). The *IP6K1* is a regulator of inositol synthesis in mammary cells (73), and its activity has been shown to promote adipose accumulation in adipose tissue (74).

The third cluster of enriched terms identified for the prioritized EBV_GWAS dataset was related to the regulation of nervous system development. Among the genes associated with the terms related to this cluster, some genes stand out as functional candidates. The *RUNX3* is a master regulator of CD4^+^/CD8^+^ T-cell lineage, and it was shown to have differentially methylated regions in CD4^+^ T cells in *Mycobacterium bovis*–infected cattle (75). Another candidate gene, the *SRF*, was described to interfere with the function of luminal cell progenitors of the mammary gland (76), which form the inner epithelial layer of the mammary ducts and alveoli, contributing to the structural integrity and function of the gland (77). The prioritized gene *TSC2* is associated with a series of biological processes relevant to a putative pleiotropic effect. The *TSC2* codifies the Tuberin 2, a negative regulator of mTORC1 (78). The mTORC1 is a major regulator of catabolism in cells, regulating several processes, such as gene transcription and protein translation (79). Regarding lactation, milk is an important activator of mTORC1, which regulates protein, lipid, and nucleotide synthesis, controlling anabolism, cell growth, and proliferation (80). In addition, the activation of mTORC1 signaling and PPARγ (via *TSC2*) is essential to adipocyte differentiation in humans (81). Interestingly, in cows, the increased milk protein synthesis caused by exogenous growth hormone is associated with mTORC1 regulation of downstream factors controlling nucleocytoplasmic export and translation of mRNA (82). The treatment with growth hormone is a well-established protocol to increase the milk protein synthesis by the ruminant mammary gland, despite all the mechanisms associated with its galactopoietic effect not being fully understood (82–85). However, there is evidence that the initiation and elongation steps of mRNA translation are affected by the administration of growth hormone during lactation (84). In the current study, two prioritized genes, *KALRN* and *ARHGEF7*, were associated with the enriched biological process “regulation of growth hormone secretion.”

Several prioritized genes, such as *SLC7A5*, *DEGS1*, *DAGLB*, *ARHGEF7*, *KALRN*, and *SERINC5*, were associated with enriched terms related to at least two functional clusters identified here. Therefore, this highlights the potential of these genes to play relevant roles in the control of the pleiotropic effect observed for the traits evaluated in the EBV_GWAS dataset.

### Prioritized genes in both datasets for the observed pleiotropic effect

The three prioritized genes for both Trait_GWAS and EBV_GWAS datasets (*PHGDH*, *SLC1A4*, and *CSN3*) play relevant roles in biological processes associated with milk production, cheese production, and resistance to mastitis. The *PHGDH* gene codifies the D-3-phosphoglycerate dehydrogenase, an enzyme responsible for serine biosynthesis. In mice, it was suggested that PHGDH-derived serine supports liver ceramide synthesis and sustains general lipid homeostasis (86). Ceramide has been identified as a potential inhibitor of insulin-stimulated glucose uptake in adipose and skeletal muscle tissues of dairy cattle (87). In addition, palmitic acid supplementation has been linked to increased ceramide production, accompanied by higher milk yield, elevated circulating nonesterified fatty acids, and enhanced adipose tissue responsiveness to glucose challenges in dairy cattle (88). Interestingly, in goats, protein deficiency in late lactation resulted in the increased expression of *PHGDH* in the liver and mammary gland compared with goats that received a standard diet (89). Therefore, this suggests a similar role of this gene in small ruminants. The *SLC1A4* is a Na-dependent neutral amino acid transporter for amino acids such as threonine, cysteine, serine, and alanine (90). The expression of *SLC1A4* decreased by half after three hours of inoculation of lipopolysaccharide from *Escherichia coli* in the mammary gland of lactating mice (91). The evaluation of amino acid transporters in the mammary gland of late lactating sows indicated that *SLC1A4*, *SLC1A5*, and *SLC38A2* exhibited relatively higher expression levels compared with other transporters, suggesting their prominent roles in amino acid transport during the milk protein synthesis process (59). In cows, the expression of *SLC1A4* was shown to be up-regulated in the mammary gland of early lactating cows compared with the late lactation stage (92). In rats, the expression of amino acid transporters varies across lactation as a response to metabolic needs, dietary protein supplies, and hormonal changes (93). Consequently, this indicates an important role of these genes during the transition from pregnancy to lactation. The *CSN3* gene codifies the κ-casein, one of the main milk proteins, responsible for playing a major role in the structural properties of milk protein concentrates (94, 95). Polymorphisms in the *CSN3* gene were widely associated with multiple milk-related traits in several species; for example, curd yield in water buffaloes (96) lactation yield, fat percentage, and protein percentage in cows (97), and milk yield, fat, protein, and lactose percentages in sheep (98, 99). Consequently, these findings place *CSN3* as one of the main functional candidate genes for a pleiotropic effect related to production traits in dairy species.

### General considerations

The gene prioritization pipeline proposed in this study is an adaptation of the methodology proposed by Wu et al. (15). Network-based gene prioritization approaches have the potential to be powerful tools for integrating multiple sources of information, enabling the construction of informative predictive models (12–14). However, some limitations must be considered in the evaluation of the efficiency of such pipelines. The current study observed a large impact on the prioritization processes caused by the absence of assigned gene symbols for several transcripts. The gene-enriched term network was based on a human database to improve the informativeness of the networks. Therefore, the absence of a gene symbol prevents the prioritization of these genes. Consequently, the availability of resources for functional annotation can directly influence the output of the pipeline. Alternative approaches, such as the annotation of GO terms and metabolic pathways through sequence homology, may help mitigate this impact. Another point to be considered is that several significant genes for the pleiotropic effects were associated with noncoding RNAs, such as long noncoding RNAs (lncRNAs). The co-expression networks analyzed in this study did not include noncoding RNAs; however, the inclusion of these molecules might improve the detection of candidate genes. It is also important to mention that despite the relatively low overlapping of the prioritized genes between the two datasets, the functional profiles of the prioritized genes, such as enriched GO terms, metabolic pathways, and QTLs, are largely shared between both lists. This low overlap in prioritized genes might be explained by the smaller number of significant genes exhibiting a pleiotropic effect in the Trait_GWAS dataset, likely due to the smaller sample size and the larger number of phenotypes. Finally, it is important to mention that the first, and probably the most crucial, part of this pipeline relies on the identification of pleiotropic signals across the genome. Here, we choose the method proposed by Bolormaa et al. (100), which is based on the integration of multi-trait statistics from individual GWAS. This method was previously used in different livestock species, such as sheep and cattle, successfully identifying candidate pleiotropic regions (101). However, it is important to mention that there are several other approaches proposed to estimate pleiotropy using genomic data. For example, Effect Direction MEta-analysis (EDME) method quantifies FDR independent of *P*-values and quantifies the pleiotropy where extreme and unevenly distributed patterns of pleiotropic effect are observed (102). The correlation scan is another interesting alternative for the identification of pleiotropic signals across the genome (103). This method uses a sliding window approach in each chromosome to identify local genomic regions that either drive or antagonize the genetic correlations between traits. Both methods, as well as others, could be used in the first step pf the proposed pipeline. Indeed, further studies evaluating the impact of different methods for estimating pleiotropic effects on the prioritization of functional candidate genes could be important to better understand the genetic architecture of genetic correlations across traits.

Moreover, the evaluation of the distribution of the significant genes based on their relevance within the networks indicated that there are specific networks where the most significant genes act as hub genes. The evaluation of the functions of these networks reveals associations with processes important for milk production and immunity, reinforcing the effectiveness of the approach applied in the current study.

Therefore, the result obtained here reinforces the applicability and effectiveness of network-based pipelines for prioritizing candidate genes displaying pleiotropic effects between milk production and mastitis resistance-related traits in dairy sheep. This pipeline could be directly applied to other livestock species for different sets of phenotypes with minimal changes. The lists of prioritized genes pinpoint relevant biological processes such as amino acid transport, lipid metabolism, regulation of mammary gland development, and control of the immune response linked to the detected pleiotropic effect. The identification of these candidate genes paves the way for further investigations of putative causal variants for the observed pleiotropic effects. This will aid in classifying the pleiotropic mechanism, relating pleiotropy to genetic correlations between traits, and applying these markers in selection programs. Further studies evaluating the inclusion of other omics data into the pipeline proposed here, such as metabolomics, proteomics, and metagenomics, could help to improve the understanding of the genetic architecture of complex traits in livestock species. Additionally, comparing different genomic selection frameworks, such as those incorporating weighting strategies for prioritized candidate genes, is necessary to properly evaluate the advantage of including markers identified in these genes into breeding programs.

## Materials and methods

### Sample, genotyping, imputation, and GWASs

The datasets used in the current study comprised 1,039 and 3,459 Assaf dairy ewes genotyped with a customized Affymetrix chip composed of 49,702 markers, here named as Trait_GWAS and EBV_GWAS, respectively. All the marker coordinates were converted to the version ARS-UI_Ramb_v2.0 of the ovine reference genome. Regarding the phenotypic data, the first dataset was composed of 12 phenotypes. A complete description of the phenotyping process for those traits is available in Marina et al. (6). The EBVs for the 3,459 Assaf dairy sheep were provided by the Spanish Assaf Sheep Breeders Association. The detailed description of the process applied to estimating the EBVs for those animals is described at a resolution that defines the national breeding program for the breed (https://www.mapa.gob.es/es/ganaderia/temas/zootecnia/razas-ganaderas/razas/catalogo-razas/ovino/assaf/datos_reglamentacion.aspx). Briefly, the EBVs for ICOp, GVFAT, GVPROT, and GV150 were obtained through the Best Linear Unbiased Predictor method using the following animal model with repeated measures:


y=RAE+TP+NL+INTP1+INTP2+g+p+e

where *y* is the target phenotype, RAE is the combined effect of flock year and lambing season, LAC is the lactation number, TP is the lambing type (single or multiple), INTP1 is the interval between lambing and the first milk control, INTP2 is the lambing interval, *g* is the additive genetic effect of the animal, *P* is the permanent environmental effect, and *e* is the residual. For GVMR, a similar model was adopted:


y=RAE+LAP+TP+INTP2+LS+g+p+e

where *y* is the somatic cell count in the milk, RAE is the combined effect of flock year and lambing season, LAP is the lactation age at lambing (which combines the number of lactations with age at lambing), TP is the lambing type, INTP2 is the lambing interval, LS is the lactation status (days between calving and the date of the milk control), *g* is the additive genetic effect of the animal, *P* is the permanent environmental effect, and *e* is the residual.

Both genotypic datasets were subjected to a quality control where markers with a minor allelic frequency (MAF) < 0.01 and a call rate < 0.95 were filtered out. In total, 50,095 and 40,854 markers were retained and used in the imputation stage for the Trait_GWAS and EBV_GWAS datasets, respectively. The reference population used for imputation was composed of 47 Assaf rams genotyped for 606,006 mapped markers. Those rams were chosen because they are used for artificial insemination and have significant descendants in the target population. The imputation was performed by the software Beagle v.5.2 (104) using the effective number of individuals in the population equal to 214 (105). Additionally, 100 iterations, with 50 burning and a sliding window of 80 cM were defined as parameters for the imputation. Finally, only markers with an allelic-*R*^2^ > 0.80, MAF > 0.01, and a call rate >0.95 were retained for the GWAS stage.

The software GCTA.V.1.94.3 (106) was used to implement a mixed linear model leaving-one-chromosome-out analysis (mlma-loco) for all the previously mentioned traits. The following model was fitted for both datasets:


$$y=a+bx+\;g^{-}+e$$

where the dataset *a* is the mean term, *b* is the additive effect of the candidate single nucleotide polymorphism (SNP) to be tested for association, *x* is the SNP genotype indicator variable coded as 0, 1, or 2, *g* is the polygenic effect for all the SNPs except those on the chromosome where the candidate SNP is located (treated as a random effect). For the Trait_GWAS dataset, initially, a null model was fitted, including the mean term; covariates (fixed effects) for days in milk, age at parturition, flock test day, and the number of lambs born; polygenic effects (random effects); and residuals (random effects). Subsequently, the adjusted phenotype was used to test the SNP association using the above-mentioned model. Regarding the EBV_GWAS, the phenotypes were fitted directly in the model without including covariates.

### Estimation of pleiotropic effect and gene-level *P*-values

The pleiotropic effects for all the markers included in the GWAS analyses were estimated using the multi-trait statistics proposed by Bolormaa et al. (100). Briefly, the multi-trait statistics were obtained as follows: *χ*^2^ = $t_{i}^{\prime }$*V*^−1^*t_i_*, where *t_i_* is the vector of signed *t*-values of SNP*_i_* for the traits, and $t_{i}^{\prime }$ is its transposed vector, *V*^−1^ is an inverse of the correlation matrix over all the traits. The multi-trait statistics was used to calculate the *P*-value for the pleiotropic effect based on a *χ*^2^ with the number of degrees of freedom equal to the number of traits. Therefore, one list of *P*-values for the pleiotropic effect was obtained for each dataset (Trait_GWAS and EBV_GWAS).

The obtained *P*-values were used to calculate gene-level *P*-values using the function gene_pval from the R package GALLO (107). In summary, the markers were first mapped following the gene coordinates available on the ARS-UI_Ramb_v2.0 genome version. The linkage disequilibrium (LD) values between the pairs of markers in each gene were estimated using the software PLINK v.1.90 (108). The LD matrix for each gene was used to obtain the eigenvalues of each mapped SNP via the prcomp function from the R software. Subsequently, the Liu et al. (109) method was applied to calculate the gene-level *P*-value, where the squared cumulative normal distribution of the *P*-values mapped within a gene and the eigenvalue for the respective SNPs were integrated.

### WGCNs and functional annotation

The transcriptome data of the milk somatic cells from 28 Assaf ewes (days 40 to 50 at first lactation) were used to obtain WGCN. The RNA extraction protocol and the bioinformatic pipeline for the mRNA mapping and quantification are described in detail by Suárez-Vega et al. (110). The pipeline for the identification of WGCN is described in detail by Fonseca et al. (111). Briefly, the read counts were normalized using Fragments Per Kilobase per Million Mapped Reads (FPKM), and gene transcripts with FPKM < 0.2 were removed from the analysis. After this step, the transcript counts were used to identify the WGCN via the getDownstreamNetwork function from the CoExpNets package (https://github.com/juanbot/CoExpNets) using 20 iterations and signed networks. The CoExpNets package is based on the WGCNA R package (112). However, in the CoExpNets pipeline, an additional step for gene reallocation using a *k*-means clustering approach is implemented (113). Finally, the adjacency matrix for each WGCN was obtained to represent the relationship among the genes within each module.

### Gene ontology, metabolic pathways, and quantitative trait loci enrichment analyses

Enrichment analyses for gene ontology (GO) and metabolic pathways were performed for the lists of genes mapped within each WGCN. The enrichment analysis for GO was performed for Biological Processes, Molecular Functions, and Cellular Components using the gost function from the gprofiler2 package in R (114). An additional step of redundancy reduction was performed using the rutils package in R (https://github.com/RHReynolds/rutils) via the Wang method with a similarity threshold of 0.7. The gost function was also used for the enrichment analysis of metabolic pathways using the KEGG and Reactome databases. For both GO and metabolic pathways, the enrichment analyses were performed using the human database as a reference. Significant enrichment was defined based on a 5% false discovery rate (5%). The QTL annotation and enrichment analysis were performed using the R package GALLO (107) for each gene with a gene-level *P*-value assigned using the Sheep QTLdb information (https://www.animalgenome.org/cgi-bin/QTLdb/OA/index). For each module, incidence matrices were created to represent the relationship between the genes and the enriched GO terms, metabolic pathways, and QTLs.

### Embedding of gene networks for dimensionality reduction

The Python library node2vec (115) was used to reduce the dimensionality of the WGCN and the incidence matrices obtained in the enrichment analyses. In this network embedding approach, initially, a weighted random walk was implemented via the BiasedRandomWalk function using 10 random walks; the probability (1/*p*) of returning to the source node equals 0.25, the probability (1/*q*) of moving away from source node equals 1, and the maximum length of a random walk equals 80. Once the sample set of walks was obtained, a low-dimensional embedding of nodes was learned using the Word2Vec approach (116). A total of 32 dimensions was defined as the output of the Word2Vec approach. Additionally, the maximum distance between the target node and its surrounding context node equals 32, and a total of 20 epochs (interactions) in the training were defined as parameters in the analyses. The Skip-Gram model, a neural network-based model, was selected to be used during the embedding by learning vector representation in the Word2Vec approach. In order to evaluate the relationship among the most significant genes in the WGCN, the weighted edge density of the top *K* genes (*K* ranges from 100 to 5,000 with step size 100) was estimated as


$$\mathrm{WE}D_{k}=\;\frac{\sum_{1}\leq i<j\leq K^{w_{ij}}}{\left(K(K-1)/2\right)},$$

where $w_{ij}$ is the edge between the genes *i* and *j* as proposed by Wu et al. (16). Regarding the representativeness of the genes in the networks composed by GO terms, metabolic pathways, and QTLs, the WED_K_ was not possible to calculate because there is not a direct connection between genes. Therefore, the mean betweenness of the top *K* genes (*K* ranges from 100 to 5,000 with step size 100) was calculated for each network (each individual co-expressed network and the GO-Gene and QTL-Gene networks). Betweenness is a centrality metric that quantifies the importance of a node in a network based on its role in facilitating communication between other nodes. Specifically, it measures how often a node lies on the shortest paths between pairs of other nodes. In the current case, this metric can indicate if the gene relates to more genes and terms, which might be relevant for genes with a higher probability to play roles in pleiotropic effects. The betweenness of a node *v* [*B*(*v*)] can be calculated a


$$B(v)=\;\underset{s\neq v\neq t}{\sum }\frac{\sigma_{st}(v)}{\sigma_{st}},$$

where *σ_st_* is the total number of shortest paths between nodes *s* and *t*, and *σ_st_*(*v*) is the number of those shortest paths that pass through node *v*.

### Representative learning and hierarchical model for gene posterior probability of association estimation

The gene-level *P*-values for the pleiotropic effect, the transcriptomic data represented by the WGCN, and the functional annotation data represented by the GO, metabolic pathway, and QTL enrichment results were integrated using an adaptation of the pipeline proposed in the REGENT (integRating Embeddings of multiple GEne NeTworks) method (16). Briefly, a hierarchical model was fitted to infer associations between genes and the potential pleiotropic effect for the traits evaluated in the Trait_GWAS and EBV_GWAS datasets. For each gene, a binary latent variable is assigned, denoting a potential association (1 denotes association, and 0 denotes absence of association). Despite the dimensionality reduction, directly incorporating the embeddings obtained in the previous steps is difficult. Therefore, a stage of representative learning was performed to convert the 32-dimensional embeddings to a one-dimensional eigenvector for each network in each dataset. First, a null model was fitted to obtain an initial PPA of each gene, resulting in soft labels for each gene. Subsequently, using linear discriminant analysis within and between classes, variances were estimated, and eigenvalues of the embedding vectors were obtained. Finally, the transpose of this vector multiplies the original embedding to compute the reduced embedding for each gene, resulting in a latent one-dimensional representation of the original embeddings. With these pieces of information, the hierarchical model is fitted using an Expectation Maximization algorithm. At this step, the model's parameters, including the PPA, are estimated. The PPA values range from 0 to 1, and a value closer to 1 suggests a higher probability of association between the gene and the pleiotropic effect for the evaluated traits. The list of prioritized genes for the Trait_GWAS and EBV_GWAS datasets was used for enrichment analyses for GO terms and metabolic pathways using the gprofiler2 package in the R software. In addition, the same lists of genes were used to perform QTL enrichment analyses using the R package GALLO. The relationship between prioritized genes and enriched QTLs was represented using the R packages igraph (117) and visNetwork (118). The betweenness of the genes in the above-mentioned networks was estimated using the betweenness function from igraph package to identify the most relevant genes.

## Supplementary Material

pgaf361_Supplementary_Data

## Data Availability

The Spanish Assaf Sheep Breeders Association (ASSAFE) is the custodian of the raw phenotype, EBVS, and genotype data of the Assaf ewes analyzed in the current study. Access to these data for research requires permission from ASSAFE under a Data Use Agreement, which can be obtained from the corresponding author or ASSAFE (https://assafe.es/contacto/). The transcriptome data analyzed during the current study are publicly available at the ArrayExpress—EMBL-EBI database under the accession number E-MTAB-13619 (https://www.ebi.ac.uk/biostudies/arrayexpress/studies/E-MTAB-13619?query=E-MTAB-13619).
